# New substitution models for rooting phylogenetic trees

**DOI:** 10.1098/rstb.2014.0336

**Published:** 2015-09-26

**Authors:** Tom A. Williams, Sarah E. Heaps, Svetlana Cherlin, Tom M. W. Nye, Richard J. Boys, T. Martin Embley

**Affiliations:** 1 Institute for Cell and Molecular Biosciences, Newcastle University, Newcastle upon Tyne NE2 4HH, UK; 2 School of Mathematics and Statistics, Newcastle University, Newcastle upon Tyne NE1 7RU, UK

**Keywords:** phylogenetics, substitution models, tree of life

## Abstract

The root of a phylogenetic tree is fundamental to its biological interpretation, but standard substitution models do not provide any information on its position. Here, we describe two recently developed models that relax the usual assumptions of stationarity and reversibility, thereby facilitating root inference without the need for an outgroup. We compare the performance of these models on a classic test case for phylogenetic methods, before considering two highly topical questions in evolutionary biology: the deep structure of the tree of life and the root of the archaeal radiation. We show that all three alignments contain meaningful rooting information that can be harnessed by these new models, thus complementing and extending previous work based on outgroup rooting. In particular, our analyses exclude the root of the tree of life from the eukaryotes or Archaea, placing it on the bacterial stem or within the Bacteria. They also exclude the root of the archaeal radiation from several major clades, consistent with analyses using other rooting methods. Overall, our results demonstrate the utility of non-reversible and non-stationary models for rooting phylogenetic trees, and identify areas where further progress can be made.

## 1. Introduction

The root of a phylogenetic tree is fundamental to its biological interpretation. For example, the eocyte hypothesis of Lake [1], in which the eukaryotic host cell emerges from within the archaeal radiation, depends critically on a root for the tree of life outside the eukaryotes or the relevant archaeal groups. And yet, phylogenies of the broadly conserved genes usually used to test hypotheses of eukaryotic origins are typically unrooted, because they are inferred under stationary, reversible substitution models in which the likelihood of the tree does not depend on the position of the root [2].

To interpret unrooted trees, biologists typically make use of external information [3]. A common strategy is to include an outgroup, or set of taxa that are known to branch outside the clade of interest (the in-group). The root can then be placed on the branch connecting the outgroup to the rest of the tree. Although widely used, outgroup rooting has the potential to interfere with the inference of in-group relationships, particularly when the outgroup is distantly related to the in-group or differs substantially in nucleotide or amino acid composition [4,5]. In such cases, outgroup rooting can lead to a phylogenetic artefact called ‘long-branch attraction’ (LBA) [6], in which fast-evolving or compositionally biased sequences are drawn towards the outgroup, appearing as basal (early diverging) members of the in-group in the inferred tree [7]. LBA is commonly invoked to explain the basal placement of the Microsporidia, a group of fast-evolving Fungi, in early eukaryotic trees [8], and it may also have played a role in the inference of the ‘three domains' tree of life, in which the eukaryotes branch as the sister group to a monophyletic Archaea [9,10].

Another limitation of standard outgroup rooting is that it cannot be used to root the tree of life, for which no obvious outgroup is known. An ingenious solution to this problem is to use pairs of paralogous genes that were already present in the last universal common ancestor (LUCA); each paralogue can then act as an outgroup to root the other. Analyses of this type have supported a root on the branch separating the Bacteria from all other cellular life [11–13], but are nonetheless fraught with difficulty [14]—as highlighted by Gouy and colleagues elsewhere in this issue [15]. In practice, it is difficult to trace gene duplications back to LUCA, so the number of genes that can be analysed is low, and the long basal branches in the resulting trees may be particularly sensitive to phylogenetic error [14].

There is therefore a clear need for alternative rooting approaches, both to corroborate results from outgroup rooting and for use in cases where outgroup rooting is problematic. Potential alternatives include the use of molecular clocks [16], gene tree parsimony [17] and, most recently, probabilistic gene tree/species tree reconciliation [18]. Here, we consider another approach to rooting phylogenetic trees: the use of non-reversible or non-stationary substitution models, in which the likelihood of the tree depends on the position of the root. These models allow the tree to be rooted as an integral part of the analysis, without the need for outgroups. Despite the potential of these approaches for addressing questions in deep phylogeny, there has been surprisingly little work on the subject. Barry & Hartigan [19] developed a non-reversible and non-stationary substitution model that was implemented by Jayaswal *et al*. [20] and has been applied to the inference of rooted trees [21]; however, the large number of parameters involved has limited the general applicability of the model. Yang & Roberts [22] proposed a non-stationary model which allowed a change in the composition vector at each speciation event. They fitted their model to small-subunit ribosomal RNA (rRNA) sequences from across the tree of life, and recovered an eocyte tree in which the root was placed between *E. coli*—the single bacterial representative—and the remaining sequences. The NDCH model of Foster [23] and the BP model of Blanquart & Lartillot [24,25] are similar except a fixed, but unknown, number of composition vectors apply to different parts of the tree. While these models have the potential to offer a more parsimonious description of the data, their unknown dimension makes model-fitting computationally difficult. Finally, Huelsenbeck *et al*. [26] investigated the ability of a non-reversible model to correctly identify the root position on simulated data and found that the approach worked best when the data contained a high degree of non-reversibility.

One reason for the lack of subsequent interest in these models may be the additional model complexity which can result from relaxing the standard assumptions of reversibility and stationarity, and the resultant increase in the computational cost of model fitting (though see [27]). Here, we describe recent advances in Bayesian modelling of non-reversible [28] and non-stationary [29] substitution processes that ameliorate some of these difficulties. We then apply these new models to root trees relating to three classical problems in evolutionary biology: the relationship between the extremophilic Bacteria *Thermus* and *Deinococcus*; the relationship of the eukaryotes to the Archaea in the ribosomal RNA tree of life and the root of the archaeal radiation.

## 2. Two models for learning about the root of a phylogenetic tree

Consider evolution at one site of a nucleotide sequence, along one branch of a phylogenetic tree. Most phylogenetic models assume that substitutions can be modelled using a continuous time Markov process (CTMP). The defining assumption which underpins these models is the Markov property which asserts that, conditional on the current state of the process (i.e. the current nucleotide), the future state depends only on this current state and not on its past. The CTMP is characterized by an instantaneous rate matrix that governs the transition probabilities along the branch. Standard models assume that the CTMP is time-reversible and in its stationary distribution. This pair of assumptions affords some mathematical simplification and allows the rate matrix to be decomposed into the probabilities of the theoretical stationary distribution and a set of exchangeability parameters [30]. The latter determine the general propensity for change between the different pairs of nucleotides. The most general form of this model, with six exchangeability parameters, is the GTR model [31]. Assumptions of equality among these parameters give rise to simpler models, such as the HKY85 model [32], which has only two: one governing the rate at which transitions occur, and the other the rate of transversions. In standard models, a rate matrix of the same form is assumed to apply to every branch of the tree. We call this assumption across-branch homogeneity. Sites are then assumed to be independent of each other and evolving according to the same model, but with a site-specific rate. The variation between rates is generally modelled using a gamma distribution or one of its discretized approximations [33].

The assumptions of stationarity, reversibility and across-branch homogeneity are largely made for mathematical convenience. However, these assumptions come at an inferential cost, with stationary and reversible models yielding likelihood functions that do not depend on the position of the root. As such, topological inference is limited to unrooted trees. We consider two recently developed Bayesian hierarchical models that relax some of these standard assumptions, and thereby allow the models to be used to make inference about root positions.

The first of these models, NR [28], is branch-homogeneous and stationary, but non-reversible. In a non-reversible model, the direction of time is important. The structure of the Bayesian hierarchical model is based on the simple HKY85 model, but allows the parameters of the instantaneous rate matrix to depart from this structure through two perturbations: the first to allow a more general GTR structure, and the second to allow the most general non-reversible form. The size of the perturbations is controlled by standard deviation parameters *σ*_R_ and *σ*_N_, respectively, whose values are unknown. By fitting the model to data, we learn which values are more or less plausible, with large values of *σ*_R_ providing evidence of reversible departures from the HKY85 model, and large values of *σ*_N_ providing evidence of non-reversibility. Indeed, it is this evidence of non-reversibility that drives inference about the root position.

The second model, HB [29], is branch-heterogeneous. Evolution on each branch proceeds according to the GTR model, but the composition vector, i.e. the theoretical stationary distribution, is allowed to take a different value on each branch that is not connected to the root. A further distinct composition vector applies at the root and on the two branches on either side. This creates a model of fixed dimension that is easier to fit than the related NDCH [23] or BP [24,25] models. To avoid overparametrization, the Bayesian hierarchical model is structured to allow information to be shared between branches, with a greater exchange of information between neighbouring than distant edges. The resulting model is non-stationary and can account for discrepancies in sequence composition among taxa. It also yields a likelihood function that is informative about the root position. In this case, the information arises from evidence of non-stationarity in the data.

Both models are fitted in a Bayesian framework, using Markov chain Monte Carlo (MCMC) methods to sample the posterior distribution. Further details of the MCMC algorithms can be found in Heaps *et al*. [29] and Cherlin *et al*. [28], although note that here we use a standard Metropolis Hastings algorithm for the HB model, rather than the data augmentation scheme described in the original paper. We also use a slightly revised version of the HB model, with a common composition vector at the root and on the two branches on either side, and GTR, rather than HKY85, exchangeabilities. Our experience suggests that this revised model provides more robustness against implausible root splits on pendant branches of the underlying unrooted tree.

## 3. The relationship between *Thermus* and *Deinococcus*

**Figure 1.**
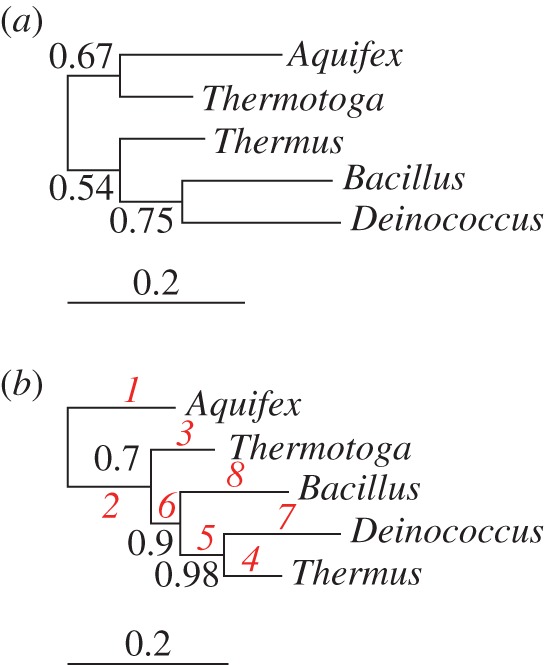
Majority rule consensus tree showing the relationship between *Thermus* and *Deinococcus* inferred under the NR and HB models. (*a*) The NR model recovers the incorrect ‘attract’ tree, in which the mesophiles (*Deinococcus* and *Bacillus*, moderate GC) and the thermophiles (*Thermus, Thermotoga* and *Aquifex*, high GC) group according to sequence composition rather than historical relatedness. (*b*) The HB model recovers the correct unrooted topology, which groups *Deinococcus* and *Thermus* to the exclusion of the other taxa. The branches are labelled in red in order of decreasing posterior mean GC content; for example, the branch leading to *Aquifex* is the most GC-rich. The root position in this tree is also consistent with a recent, broadly sampled phylogenomic survey of bacterial diversity [38], and the corresponding root split received the most posterior support under both models (electronic supplementary material, table S1, PP = 0.21 under NR, PP = 0.70 under HB). The HB model probably performs better than the NR model on this dataset because it is able to account for the compositional shift to high GC that occurred in *Thermus* following the split from *Deinococcus.* Support values are given as Bayesian posterior probabilities, and branch lengths are proportional to the expected number of substitutions per site, as indicated by the scale bar.

*Deinococcus radiodurans* and *Thermus aquaticus* are related extremophilic Bacteria that are adapted to two different sets of extreme conditions: ionizing radiation and desiccation in the case of *Deinococcus* [34], and high temperatures for *Thermus* [35]. Although their common ancestry is attested by a range of independent analyses [36], the *Thermus*–*Deinococcus* relationship is a classic test case for new phylogenetic models because standard approaches often fail to recover the correct tree from analyses of rRNA [23,37]. To compare the behaviour of the NR and HB models, we inferred trees under both models using an alignment [23] of the 16S rRNA genes of *Thermus*, *Deinococcus* and three outgroup taxa: *Bacillus, Aquifex* and *Thermotoga*. The majority rule consensus tree under the NR model represents an incorrect tree (figure 1*a*), with *Bacillus* as the closest relative of *Deinococcus.* This tree recapitulates previous results in which the rRNA sequences cluster according to GC content rather than evolutionary history: *Thermus*, *Aquifex* and *Thermotoga* all have GC-rich rRNA genes, perhaps as an adaptation to life at high temperatures [39], while both *Deinococcus* and *Bacillus* are mesophiles whose rRNA genes show a more moderate GC content. The consensus tree inferred under the HB model correctly recovered the *Thermus*/*Deinococcus* clade (figure 1*b*), probably because the HB model allows composition to vary across the branches of the tree. The numbered ranking of branches in order of decreasing GC content in figure 1*b* demonstrates that placing *Thermus* and *Deinococcus* as sister taxa requires a switch to high GC-content in *Thermus* following the divergence of the two lineages, which is not possible under stationary models such as NR.

Although the consensus trees in figure 1 disagree on the position of the root, it is important to note that the root position in a consensus tree does not necessarily represent the root split which received the most posterior support. This is because the majority rule consensus tree contains precisely the clades that have posterior support of more than 0.5 [40]. It is therefore a conditional summary, computed recursively from the leaves to the root, which depends upon the plausibility of sub-clades. By contrast, the posterior over root splits is a marginal summary which averages over the relationships expressed elsewhere in the tree (see [28] for further details or [41] for related comments on summarizing posteriors for unrooted topologies). Therefore, in spite of the differences between the consensus trees in figure 1, it is interesting that the root split which received the most support was the same in both cases, and separated *Aquifex* from the other species (electronic supplementary material, table S1, PP = 0.21 under NR, PP = 0.70 under HB). This result is consistent with the conclusions of the Genomic Encyclopedia of Bacteria and Archaea project, which performed the most comprehensive phylogenomic survey of Bacteria to date [38]. Thus, despite the differences between the NR and HB models in the way that rooting information is extracted from the data, both models agree with recent biological opinion in this case.

## 4. Application to the ribosomal RNA tree of life

**Figure 2.**
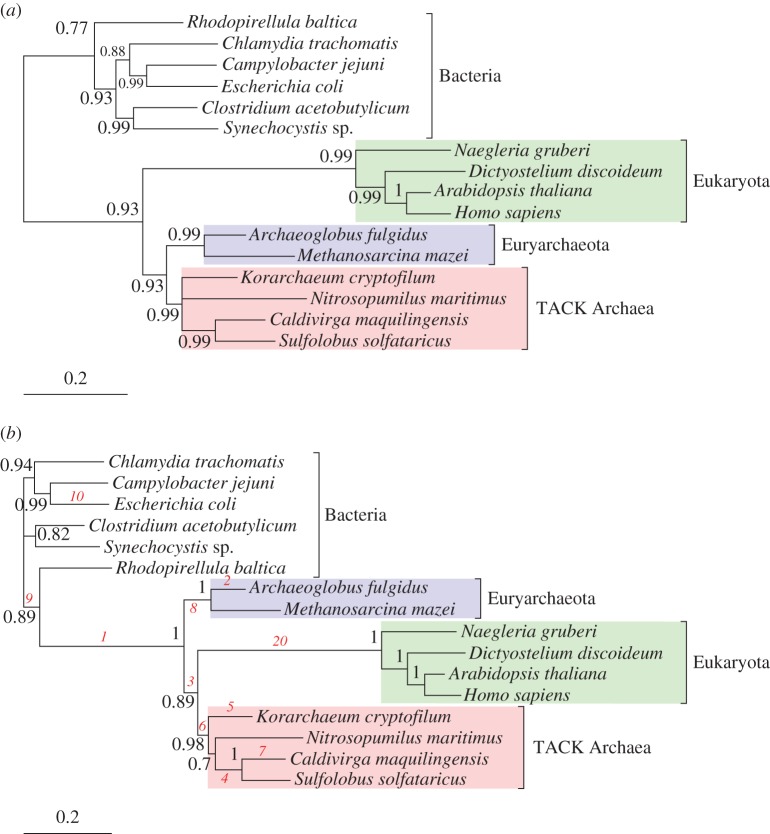
Compositional shifts in rRNA during the evolution of Bacteria, Archaea and eukaryotes. (*a*) On these 16 taxa, inference under the NR model recovers a three domains tree in which the eukaryotes form the sister group to a monophyletic Archaea (PP = 0.93). The root in the consensus tree lies on the branch connecting the Bacteria to all other cells, consistent with analyses of anciently duplicated genes [11–13]. (*b*) Inference under the HB model recovers a tree consistent with the ‘eocyte’ hypothesis, in which the eukaryotic rRNA sequences emerge from within the Archaea. This relationship is supported by analyses of rRNA that include more taxa and analyses of broadly conserved protein-coding genes [10,43,52]. The branches are labelled in red in order of decreasing posterior mean GC content; for example, the branch leading to the common ancestor of the Archaea is the most GC-rich in the tree. Support values are given as Bayesian posterior probabilities, and branch lengths are proportional to the expected number of substitutions per site, as indicated by the scale bar.

Comparisons of rRNA sequences have been central to the debates over the deep structure of the tree of life, and in particular the relationship of eukaryotes to the Archaea [10]. Many early analyses favoured a ‘three domains’ tree, in which the Bacteria, Archaea and eukaryotes were each monophyletic domains. By contrast, more recent analyses taking advantage of an improved sampling of archaeal sequence diversity and using better-fitting substitution models have instead favoured the ‘eocyte’ tree of Lake [1], in which the eukaryotic rRNA sequences—taken to represent the host cell lineage for the mitochondrial endosymbiont—emerge from within the archaeal radiation [42–45]. We analysed a previously published 16-species concatenated rRNA alignment containing 761 sites from the large subunit rRNA gene and 720 sites from the small subunit [29]. Sequences were aligned with MUSCLE [46], MAFFT [47], ProbCons [48] and Kalign [49], and a consensus alignment inferred using M-Coffee [50]. Poorly aligning sites were identified and removed using BMGE [51] with the default parameters. Analysis of this alignment under the NR model recovered the classic ‘three domains’ topology, in which the eukaryotes emerge as the sister group to a monophyletic Archaea with strong posterior support (figure 2*a*, PP = 0.93 for archaeal monophyly). Based on recently published analyses of rRNA and protein-coding genes, this tree is currently thought to be incorrect [10,42,44,52,53], although it has historically received support from simpler stationary models (reviewed in [10]). This result suggested that, while the NR model can provide useful rooting information, it is subject to many of the same limitations as other stationary models. Inference under the HB model recovered an eocyte tree, with the eukaryotes emerging as the sister group to the ‘TACK’ superphylum of Archaea (figure 2*b*, PP = 0.89 for the eukaryote/TACK clade), consistent with recent phylogenomic analyses [42–45]. As in the case of the *Thermus* dataset, mapping posterior inferences of the most GC-rich branches onto the consensus tree provides an intuitive explanation for the differences in results between the NR and HB models. The branches leading to the common ancestor of the Archaea, and to each of the major archaeal clades, are among the most GC-rich in the phylogeny (ranked first (0.756), sixth (0.639) and eighth (0.621) by posterior mean GC content; see figure 2*b* and electronic supplementary material, figure S2), but the long branch leading to the common ancestor of the eukaryotes has a much more moderate GC content (ranked 20th overall; 0.444). Thus, the eocyte tree requires the placement of a moderate GC branch inside a high GC clade: this is biologically plausible, because we know that sequence composition can change over evolutionary time, but not possible under NR and other stationary models. This result provides some insight into why early analyses with simpler phylogenetic methods often recovered the three domains tree and provides support for the suggestion of Tourasse & Gouy [9] that the eocyte tree might be intrinsically more difficult to recover than the three domains tree.

**Figure 3.**
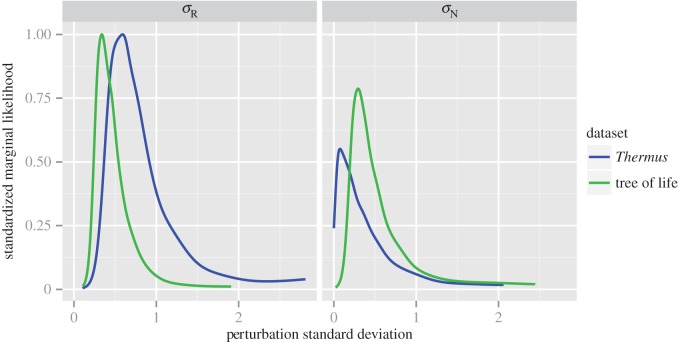
Evidence of GTR-like structure and non-reversibility in the *Thermus–Deinococcus* and tree of life datasets. The plots show the standardized marginal likelihood (proportional to the posterior density divided by the prior density) for the reversible (*σ*_R_) and non-reversible (*σ*_N_) perturbation standard deviation parameters of the NR model. They summarize the weight of evidence from the data for different values of *σ*_R_ and *σ*_N_ given the choice of model and prior. (*a*) There is strong evidence of non-zero *σ*_R_ for both datasets, suggesting that GTR exchangeabilities are more plausible than HKY85 exchangeabilities in both cases. (*b*) There is also evidence for small, but non-zero, values of *σ*_N_ in both datasets, which provides evidence of detectable non-reversibility in the data. This is particularly true for the tree of life dataset, which may partially explain the failure of the HB model to recover the most widely accepted root position (i.e. between the Bacteria and Archaea).

For these rRNA genes, the posterior distributions for root splits support the placement of the roots on the consensus trees, showing disagreement between the NR and HB models (see figure 2 and electronic supplementary material, table S2). The NR model favours a root on the branch separating the Bacteria from all other cells, in agreement with traditional paralogue rooting approaches [11–13] and analyses of genome networks [54]. By contrast, the HB model places the root *within* the Bacteria (figure 2*b*) with posterior support equal to 1. Although the root is unresolved on the consensus tree, the root split with the greatest posterior support (electronic supplementary material, table S2, PP = 0.34) groups all the Bacteria except *Rhodopirellula* on one side of the root. Some authors have argued for a root within the Bacteria on the basis of polarized indels or other rare genomic changes [55,56], although neither of these proposals unites the planctomycetes (here represented by *Rhodopirellula*) with the Archaea and eukaryotes. While resolution of this issue will clearly require analyses with a greatly improved sampling of Bacteria, we also sought to investigate the reason for the different root inferences under the NR and HB models. Recall that, in the case of the NR model, the *σ*_R_ parameter provides a measure of reversible departures from the HKY85 model while the *σ*_N_ parameter provides a measure of non-reversibility. Plots showing the weight of evidence in the data for different values of *σ*_N_ and *σ*_R_ for the *Thermus* and tree of life datasets showed markedly different behaviour (figure 3): while both datasets revealed evidence of non-zero values for *σ*_R_, providing support for GTR-like over HKY85 exchangeabilities, posterior support for non-zero values of *σ*_N_ is clearly greater in the tree of life. Thus, the tree of life dataset shows substantial evidence of non-reversibility in the substitution process *within* branches, which is not accounted for in the HB model. This observation may provide a partial explanation for the failure of the HB model to recover the most widely accepted root position on this dataset. It also suggests that, beyond the compositional heterogeneity that is increasingly recognized as an important and pervasive feature of real sequence data, some alignments may also contain significant evidence of non-reversibility in the substitution process. This finding agrees with the work of Squartini & Arndt [57], who presented evidence for non-reversibility in the evolution of the *Drosophila* and human lineages, and motivates the development of phylogenetic models that can account for both non-stationarity and non-reversibility, as these may both be salient features of real sequence data.

## 5. The root of the archaeal radiation

**Figure 4.**
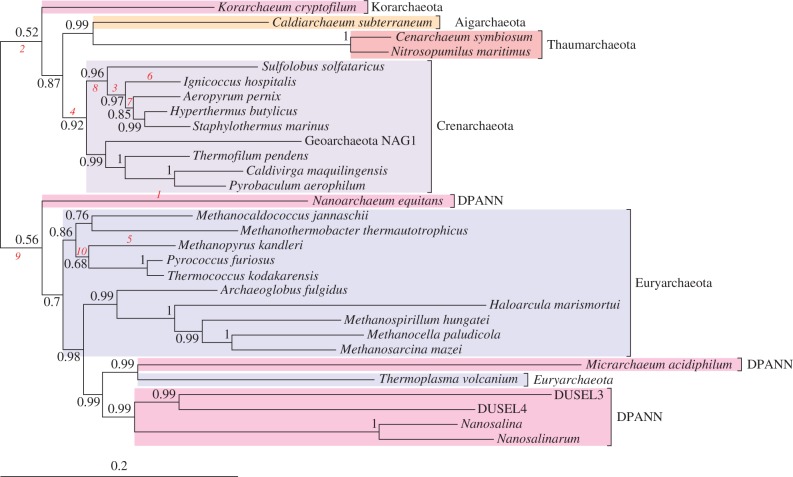
The root of the Archaea under the HB model. The consensus tree places the archaeal root between the ‘TACK’ Archaea and a clade containing the Euryarchaeota, from within which the DPANN lineages emerge polyphyletically. Posterior support for this root split is relatively low (electronic supplementary material, table S3, PP = 0.07), and our analysis does not exclude the root from some neighbouring branches, including *Korarchaeum*, *Nanoarchaeum*, or some of the early diverging methanogenic lineages within the Euryarchaeota. Branches are labelled in order of decreasing GC content; for clarity, only the 10 most GC-rich branches are labelled. Support values are provided as Bayesian posterior probabilities, and branch lengths are proportional to the expected number of substitutions per site, as indicated by the scale bar.

If the root of the tree of life lies between the Bacteria and Archaea, then the divergence of these two lineages represents the deepest split in the cellular world. Rooting the archaeal tree would establish a phylogenetic framework for reconstructing the gene content of the first archaeon and for constraining hypotheses about the earliest archaeal metabolisms. The models we introduce here are particularly well suited to addressing this question because they obviate the need to include a bacterial outgroup and the long branch that results, potentially improving the robustness of our inferences against long-branch attraction. We considered a concatenated alignment of 16S and 23S rRNA sequences sampled from across the known diversity of Archaea, including the Euryarchaeota, ‘TACK’ superphylum [45], and recently described ‘DPANN’ lineages [38]. The archaeal alignment showed substantially more heterogeneity across taxa in its empirical composition than the *Thermus* or tree of life datasets. For example, the standard deviation of the proportion of guanine was 0.064, compared with at most 0.027 for the other two alignments (electronic supplementary material, table S4). The stationary NR model cannot account for such compositional heterogeneity and so we chose to fit the HB model only to this dataset. The root in the consensus tree separates the ‘TACK’ superphylum from a clade containing the Euryarchaeota and ‘DPANN’ Archaea (figure 4), although posterior support for the monophyly of the clades on either side of this root was low (PP = 0.52 and 0.56, respectively), largely due to uncertainty about the position of *Korarchaeum* on one side of the root, and of *Nanoarchaeum* and some early diverging methanogenic Euryarchaeota on the other. This uncertainty is reflected in the posterior for root splits, which offers support to a variety of basal arrangements of these groups around the root; see the electronic supplementary material, table S3. It is also interesting to note that the most GC-rich branches in the consensus tree (those leading to *Nanoarchaeum* on the one hand, and the TACK superphylum on the other) are close to the inferred root, consistent with our analysis of the rRNA tree of life (figure 2*b*). Based on the observation that the GC content of rRNA genes increases with optimal growth temperature [39], these results are compatible with the idea that the archaeal common ancestor was a thermophile [58]. Intriguingly, the analysis robustly excluded the root from within several major archaeal groups, including the Crenarchaeota (PP = 0.92), Thaumarchaeota/Aigarchaeota (PP = 0.99), and a clade containing some Euryarchaeota and all of the ‘DPANN’ Archaea except *Nanoarchaeum* (PP = 0.98).

A key benefit of the HB model is that it allows inference about the root of the tree without the use of an outgroup while directly modelling the variation of sequence composition over time. It is therefore interesting to note that our rooted archaeal tree shares some key features with a recent phylogenomic analysis of the Archaea that made use of proteins for which the distance separating bacterial and archaeal sequences was shorter than in traditional ribosomal protein trees, an approach which should also reduce the impact of long-branch attraction on the in-group phylogeny [59]. In both trees, the highly reduced ‘DPANN’ Archaea emerge polyphyletically from within the Euryarchaeota, and the root is placed between TACK (called ‘Proteoarchaeota’ in [59]) and this Euryarchaeota/DPANN clade. Taken together, these results suggest that the basal position of a monophyletic DPANN clade in recent analyses may, in part, be attributable to attraction to the long bacterial outgroup. This result illustrates how the approaches described here can complement and extend analyses using traditional outgroup rooting.

## 6. Prospects for non-reversible and non-stationary substitution models

Despite their potential for addressing key questions in early cellular evolution, non-reversible and non-stationary substitution models are still an under-explored area of phylogenetics. In this article, we have explored two recently developed models for inferring rooted trees from nucleotide data on modest but reasonable numbers of taxa—up to 30 in the case of the archaeal dataset. These models make root inferences that are consistent with independent phylogenomic analyses and anciently duplicated genes and may provide a useful alternative to outgroup rooting. Our results therefore show that real sequence alignments can contain useful information about the position of the root that is implicit both in changes in sequence composition over time as well as—in at least some cases—evidence for directionality in the process of substitution.

Inferring deep phylogenies is challenging, and our analyses also revealed limitations in the models we have developed so far, helping to identify some important points on which progress can be made. Compositional heterogeneity is a pervasive feature of real sequence data, but at least some alignments also show evidence for non-reversibility within branches of the tree (that is, *σ*_N_ > 0). Joint modelling of these features is desirable, but inference under such models is not yet computationally tractable. We have focused on relaxing modelling assumptions so as to make the likelihood dependent on the root of the tree, but we know that other model properties are also important for the accuracy of the inferred topology—in particular, across-site compositional variation (as accommodated by the CAT model [60]). Future work will focus on incorporating these and other important features into our models, in order to improve the accuracy and robustness of rooted phylogenetic trees.

## Supplementary Material

Electronic supplementary material

## Data accessibility

The sequence alignments analysed in the paper have been deposited at FigShare: http://dx.doi.org/10.6084/m9.figshare.1468407.

## References

[1] Lake JA, Henderson E, Oakes M, Clark MW 1984 Eocytes: a new ribosome structure indicates a kingdom with a close relationship to eukaryotes. Proc. Natl Acad. Sci. USA 81, 3786–3790. (10.1073/pnas.81.12.3786) 6587394 PMC345305

[2] Felsenstein J 1981 Evolutionary trees from DNA sequences: a maximum likelihood approach. J. Mol. Evol. 17, 368–376. (10.1007/BF01734359) 7288891

[3] Penny D 1976 Criteria for optimising phylogenetic trees and the problem of determining the root of a tree. J. Mol. Evol. 8, 95–116. (10.1007/BF01739097) 966292

[4] Tarrío R, Rodríguez-Trelles F, Ayala FJ 2000 Tree rooting with outgroups when they differ in their nucleotide composition from the ingroup: the *Drosophila saltans* and *willistoni* groups, a case study. Mol. Phylogenet. Evol. 16, 344–349. (10.1006/mpev.2000.0813) 10991788

[5] Holland BR, Penny D, Hendy MD 2003 Outgroup misplacement and phylogenetic inaccuracy under a molecular clock—a simulation study. Syst. Biol. 52, 229–238. (10.1080/10635150390192771) 12746148

[6] Felsenstein J 1978 Cases in which parsimony or compatibility methods will be positively misleading. Syst. Zool. 27, 401–410. (10.2307/2412923)

[7] Philippe H, Laurent J 1998 How good are deep phylogenetic trees? Curr. Opin. Genet. Dev. 8, 616–623. (10.1016/S0959-437X(98)80028-2) 9914208

[8] Hirt RP, Logsdon JM, Healy B, Dorey MW, Doolittle WF, Embley TM 1999 Microsporidia are related to Fungi: evidence from the largest subunit of RNA polymerase II and other proteins. Proc. Natl Acad. Sci. USA 96, 580–585. (10.1073/pnas.96.2.580) 9892676 PMC15179

[9] Tourasse NJ, Gouy M 1999 Accounting for evolutionary rate variation among sequence sites consistently changes universal phylogenies deduced from rRNA and protein-coding genes. Mol. Phylogenet. Evol. 13, 159–168. (10.1006/mpev.1999.0675) 10508549

[10] Williams TA, Foster PG, Cox CJ, Embley TM 2013 An archaeal origin of eukaryotes supports only two primary domains of life. Nature 504, 231–236. (10.1038/nature12779) 24336283

[11] Iwabe N, Kuma K, Hasegawa M, Osawa S, Miyata T 1989 Evolutionary relationship of archaebacteria, eubacteria, and eukaryotes inferred from phylogenetic trees of duplicated genes. Proc. Natl Acad. Sci. USA 86, 9355–9359. (10.1073/pnas.86.23.9355) 2531898 PMC298494

[12] Gogarten JP et al. 1989 Evolution of the vacuolar H+-ATPase: implications for the origin of eukaryotes. Proc. Natl Acad. Sci. USA 86, 6661–6665. (10.1073/pnas.86.17.6661) 2528146 PMC297905

[13] Brown JR, Doolittle WF 1995 Root of the universal tree of life based on ancient aminoacyl-tRNA synthetase gene duplications. Proc. Natl Acad. Sci. USA 92, 2441–2445. (10.1073/pnas.92.7.2441) 7708661 PMC42233

[14] Philippe H, Forterre P 1999 The rooting of the universal tree of life is not reliable. J. Mol. Evol. 49, 509–523. (10.1007/PL00006573) 10486008

[15] Gouy R, Baurain D, Philippe H 2015 Rooting the tree of life: the phylogenetic jury is still out. Phil. Trans. R. Soc. B 370, 20140329 (10.1098/rstb.2014.0329) 26323760 PMC4571568

[16] Drummond AJ, Ho SYW, Phillips MJ, Rambaut A 2006 Relaxed phylogenetics and dating with confidence. PLoS Biol. 4, 699–710. (10.1371/journal.pbio.0040088) PMC1395354 16683862

[17] Katz LA, Grant JR, Parfrey LW, Burleigh JG 2012 Turning the crown upside down: gene tree parsimony roots the eukaryotic tree of life. Syst. Biol. 61, 653–660. (10.1093/sysbio/sys026) 22334342 PMC3376375

[18] Boussau B, Szöllösi G, Duret L 2013 Genome-scale coestimation of species and gene trees. Genome Res. 23, 323–330. (10.1101/gr.141978.112) 23132911 PMC3561873

[19] Barry D, Hartigan J 1987 Statistical analysis of hominoid molecular evolution. Stat. Sci. 2, 191–210. (10.1214/ss/1177013353)

[20] Jayaswal V, Jermiin LS, Robinson J 2005 Estimation of phylogeny using a general Markov model. Evol. Bioinform. Online 1, 62–80. PMC2658871 19325854

[21] Jayaswal V, Ababneh F, Jermiin LS, Robinson J 2011 Reducing model complexity of the general Markov model of evolution. Mol. Biol. Evol. 28, 3045–3059. (10.1093/molbev/msr128) 21593046

[22] Yang Z, Roberts D 1995 On the use of nucleic acid sequences to infer early branchings in the tree of life. Mol. Biol. Evol. 12, 451–458. 7739387 10.1093/oxfordjournals.molbev.a040220

[23] Foster P 2004 Modeling compositional heterogeneity. Syst. Biol. 53, 485–495. (10.1080/10635150490445779) 15503675

[24] Blanquart S, Lartillot N 2006 A Bayesian compound stochastic process for modeling nonstationary and nonhomogeneous sequence evolution. Mol. Biol. Evol. 23, 2058–2071. (10.1093/molbev/msl091) 16931538

[25] Blanquart S, Lartillot N 2008 A site- and time-heterogeneous model of amino acid replacement. Mol. Biol. Evol. 25, 842–858. (10.1093/molbev/msn018) 18234708

[26] Huelsenbeck JP, Bollback JP, Levine AM 2002 Inferring the root of a phylogenetic tree. Syst. Biol. 51, 32–43. (10.1080/106351502753475862) 11943091

[27] Boussau B, Gouy M 2006 Efficient likelihood computations with nonreversible models of evolution. Syst. Biol. 55, 756–768. (10.1080/10635150600975218) 17060197

[28] Cherlin S, Nye TMW, Boys RJ, Heaps SE, Williams TA, Embley TM 2015 The effect of non-reversibility on inferring rooted phylogenies. See http://arxiv.org/pdf/1505.08009.pdf. 10.1093/molbev/msx294 PMC5889004 29149300

[29] Heaps SE, Nye TMW, Boys RJ, Williams TA, Embley TM 2014 Bayesian modelling of compositional heterogeneity in molecular phylogenetics. Stat. Appl. Genet. Mol. Biol. 13, 589–609. (10.1515/sagmb-2013-0077) 25153609

[30] Whelan S, Goldman N 2001 A general empirical model of protein evolution derived from multiple protein families using a maximum-likelihood approach. Mol. Biol. Evol. 18, 691–699. (10.1093/oxfordjournals.molbev.a003851) 11319253

[31] Tavaré S 1986 Some probabilistic and statistical problems in the analysis of DNA sequences. In Lectures on mathematics in the life sciences, vol. 17, pp. 57–86. Providence, RI: American Mathematical Society.

[32] Hasegawa M, Kishino H, Yano T 1985 Dating of the human–ape splitting by a molecular clock of mitochondrial DNA. J. Mol. Evol. 22, 160–174. (10.1007/BF02101694) 3934395

[33] Yang Z 1994 Maximum likelihood phylogenetic estimation from DNA sequences with variable rates over sites: approximate methods. J. Mol. Evol. 39, 306–314. (10.1007/BF00160154) 7932792

[34] Cox MM, Battista JR 2005 *Deinococcus radiodurans*—the consummate survivor. Nat. Rev. Microbiol. 3, 882–892. (10.1038/nrmicro1264) 16261171

[35] Brock TD, Freeze H 1969 *Thermus aquaticus* gen. n. and sp. n., a nonsporulating extreme thermophile. J. Bacteriol. 98, 289–297. 5781580 10.1128/jb.98.1.289-297.1969 PMC249935

[36] Omelchenko MV, Wolf YI, Gaidamakova EK, Matrosova VY, Vasilenko A, Zhai M, Daly MJ, Koonin EV, Makarova KS 2005 Comparative genomics of *Thermus thermophilus* and *Deinococcus radiodurans*: divergent routes of adaptation to thermophily and radiation resistance. BMC Evol. Biol. 5, 57 (10.1186/1471-2148-5-57) 16242020 PMC1274311

[37] Embley TM, Thomas RH, Williams RAD 1993 Reduced thermophilic bias in the 16S rDNA sequence from *Thermus ruber* provides further support for a relationship between *Thermus* and *Deinococcus*. Syst. Appl. Microbiol. 16, 25–29. (10.1016/S0723-2020(11)80247-X)

[38] Rinke C et al. 2013 Insights into the phylogeny and coding potential of microbial dark matter. Nature 499, 431–437. (10.1038/nature12352) 23851394

[39] Galtier N, Lobry JR 1997 Relationships between genomic G+C content, RNA secondary structures, and optimal growth temperature in prokaryotes. J. Mol. Evol. 44, 632–636. (10.1007/PL00006186) 9169555

[40] Bryant D 2003 A classification of consensus methods for phylogenetics. DIMACS Ser. Discret. Math. Theor. Comput. Sci. 61, 163–184.

[41] Cranston KA, Rannala B 2007 Summarizing a posterior distribution of trees using agreement subtrees. Syst. Biol. 56, 578–590. (10.1080/10635150701485091) 17654363

[42] Williams TA, Foster PG, Nye TMW, Cox CJ, Embley TM 2012 A congruent phylogenomic signal places eukaryotes within the Archaea. Proc. R. Soc. B 279, 4870–4879. (10.1098/rspb.2012.1795) PMC3497233 23097517

[43] Williams TA, Embley TM 2014 Archaeal ‘dark matter’ and the origin of eukaryotes. Genome Biol. Evol. 6, 474–481. (10.1093/gbe/evu031) 24532674 PMC3971582

[44] Lasek-Nesselquist E, Gogarten JP 2013 The effects of model choice and mitigating bias on the ribosomal tree of life. Mol. Phylogenet. Evol. 69, 17–38. (10.1016/j.ympev.2013.05.006) 23707703

[45] Guy L, Ettema TJG 2011 The archaeal ‘TACK’ superphylum and the origin of eukaryotes. Trends Microbiol. 19, 580–587. (10.1016/j.tim.2011.09.002) 22018741

[46] Edgar RC 2004 MUSCLE: multiple sequence alignment with high accuracy and high throughput. Nucleic Acids Res. 32, 1792–1797. (10.1093/nar/gkh340) 15034147 PMC390337

[47] Katoh K, Kuma K, Toh H, Miyata T 2005 MAFFT version 5: improvement in accuracy of multiple sequence alignment. Nucleic Acids Res. 33, 511–518. (10.1093/nar/gki198) 15661851 PMC548345

[48] Do CB, Mahabhashyam MSP, Brudno M, Batzoglou S 2005 ProbCons: Probabilistic consistency-based multiple sequence alignment. Genome Res. 15, 330–340. (10.1101/gr.2821705) 15687296 PMC546535

[49] Lassmann T, Sonnhammer ELL 2005 Kalign—an accurate and fast multiple sequence alignment algorithm. BMC Bioinform. 6, 298 (10.1186/1471-2105-6-298) PMC1325270 16343337

[50] Wallace IM, O'Sullivan O, Higgins DG, Notredame C 2006 M-Coffee: combining multiple sequence alignment methods with T-Coffee. Nucleic Acids Res. 34, 1692–1699. (10.1093/nar/gkl091) 16556910 PMC1410914

[51] Criscuolo A, Gribaldo S 2010 BMGE (Block Mapping and Gathering with Entropy): a new software for selection of phylogenetic informative regions from multiple sequence alignments. BMC Evol. Biol. 10, 210 (10.1186/1471-2148-10-210) 20626897 PMC3017758

[52] Cox CJ, Foster PG, Hirt RP, Harris SR, Embley TM 2008 The archaebacterial origin of eukaryotes. Proc. Natl Acad. Sci. USA 105, 20 356–20 361. (10.1073/pnas.0810647105) 19073919 PMC2629343

[53] Foster PG, Cox CJ, Embley TM 2009 The primary divisions of life: a phylogenomic approach employing composition-heterogeneous methods. Phil. Trans. R. Soc. B 364, 2197–2207. (10.1098/rstb.2009.0034) 19571240 PMC2873002

[54] Dagan T, Roettger M, Bryant D, Martin W 2010 Genome networks root the tree of life between prokaryotic domains. Genome Biol. Evol. 2, 379–392. (10.1093/gbe/evq025) 20624742 PMC2997548

[55] Cavalier-Smith T 2006 Rooting the tree of life by transition analyses. Biol. Direct 1, 19 (10.1186/1745-6150-1-19) 16834776 PMC1586193

[56] Lake JA, Skophammer RG, Herbold CW, Servin JA 2009 Genome beginnings: rooting the tree of life. Phil. Trans. R. Soc. B 364, 2177–2185. (10.1098/rstb.2009.0035) 19571238 PMC2873003

[57] Squartini F, Arndt PF 2008 Quantifying the stationarity and time reversibility of the nucleotide substitution process. Mol. Biol. Evol. 25, 2525–2535. (10.1093/molbev/msn169) 18682605

[58] Groussin M, Gouy M 2011 Adaptation to environmental temperature is a major determinant of molecular evolutionary rates in Archaea. Mol. Biol. Evol. 28, 2661–2674. (10.1093/molbev/msr098) 21498602

[59] Petitjean C, Deschamps P, López-García P, Moreira D 2014 Rooting the domain Archaea by phylogenomic analysis supports the foundation of the new kingdom Proteoarchaeota. Genome Biol. Evol. 7, 191–204. (10.1093/gbe/evu274) 25527841 PMC4316627

[60] Lartillot N, Philippe H 2004 A Bayesian mixture model for across-site heterogeneities in the amino-acid replacement process. Mol. Biol. Evol. 21, 1095–1109. (10.1093/molbev/msh112) 15014145

