# Supplementary material for: New substitution models for rooting phylogenetic trees
Source: Philos Trans R Soc Lond B Biol Sci. 2015 Sep 26;370(1678):20140336. doi: 10.1098/rstb.2014.0336 (PMC4571574; doi:10.1098/rstb.2014.0336)
Supplement: Electronic supplementary material [file rstb20140336supp1.pdf]

Electronic supplementary material for "New substitution models for rooting phylogenetic trees"

Tom A. Williams[1]*, Sarah E. Heaps[1,2], Svetlana Cherlin[1,2], Tom M.W. Nye[2], Richard J. Boys[2], T. Martin Embley[1]

*Corresponding author: tom.williams2@ncl.ac.uk

1. Institute for Cell and Molecular Biosciences, Newcastle University, Newcastle upon Tyne, NE2 4HH, UK.
2. School of Mathematics and Statistics, Newcastle University, Newcastle upon Tyne, NE1 7RU, UK.

Supplementary Text

Choice of prior

For the HB analyses we used a very similar prior specification to that described in [1]. In brief, this involved independent gamma Ga(1,1) priors for the five non-fixed exchangeability parameters, independent exponential Exp(10) priors for the branch lengths and a Gamma(10, 10) prior for the shape parameter in the discrete gamma model for across site rate heterogeneity. In the conditional prior for the branch compositions, given the topology, we chose the hyperparameters to be $a_\beta = 0.85$ and $b_\beta = 0.47$ for the *Thermus* dataset, and $a_\beta = 0.94$ and $b_\beta = 0.31$ for the tree of life and Archaea datasets. The justification for these choices can be found in [1]. For the rooted topology, we specified a Yule prior. This is described fully in [2] but, crucially, compared with a uniform prior over rooted topologies, the Yule prior offers less support to trees rooted on pendant edges, in keeping with our prior beliefs that root positions are likely to be deeper in the tree.

For the NR model, we used an identical prior specification for the branch lengths, rooted topology and shape parameter in the discrete gamma model for across site rate

heterogeneity. In the prior for the off-diagonal elements of the rate matrix, we used the hierarchical specification outlined in [3]. For the unknown parameters in this hierarchical specification, we chose, independently, a Dirichlet Dir(1,1,1,1) prior for the composition vector, a lognormal LN(0, 0.37) prior for the transition-transversion rate ratio and exponential Exp(2.3) priors for the perturbation standard deviations, σ_R and σ_N.

Markov Chain Monte Carlo scheme

We used the MCMC algorithm described in Cherlin et al. (2015) to fit the NR model to the alignments. For the HB model, we used a modification of the algorithm described in [1], omitting the data augmentation of substitutional histories, and using Metropolis Hastings updates for all parameters. In each case, we generated 1M draws from the posterior, after a burn-in period of at least 10K samples, thinning the output to retain every 100th iterate. We diagnosed convergence of the MCMC samplers using the procedure described in [1]. This involved running two chains, initialised at different starting points, and examining diagnostic plots based on both model parameters and clade frequencies. None of the checks gave evidence of any lack of convergence.

Supplementary Figures

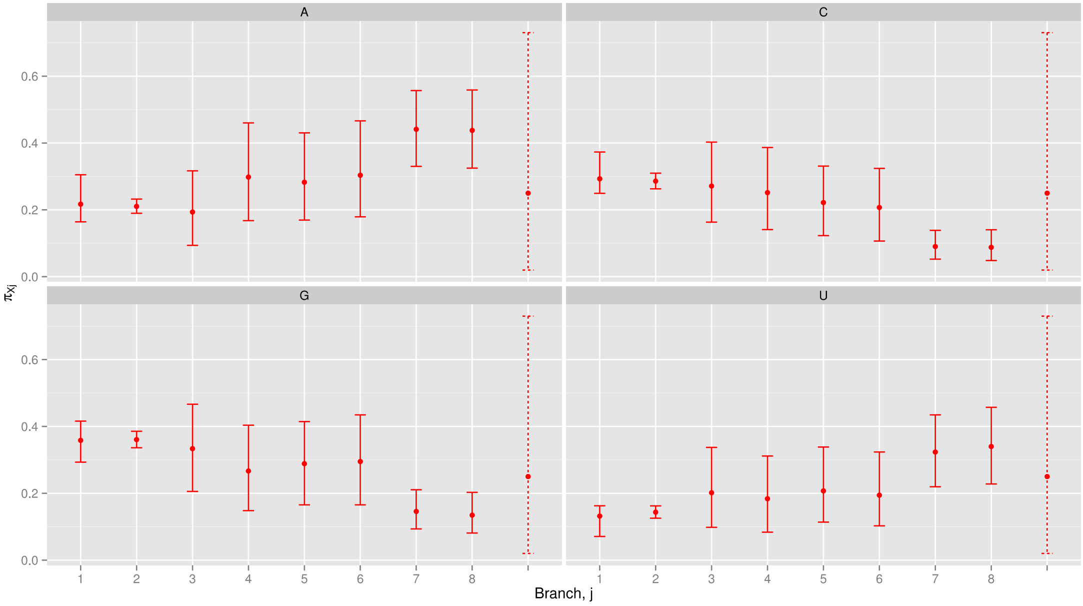

Supplementary Figure 1: Posterior inferences of nucleotide composition on each branch of the *Thermus-Deinococcus* consensus tree inferred under the HB model. The posterior summaries are based on the consensus tree and branch labels in Figure 1(b) of the main text in which the edges are labelled in decreasing order of posterior mean GC content. For every labelled branch, the plots show the mean, 2.5% point and 97.5% point in the posterior distribution for each element in the associated composition vector. Each branch was identified with the descendant clade and posteriors were computed numerically from the MCMC output by averaging the composition vectors over all draws in which the sampled tree contained that clade. The arrows with dashed lines show the mean, 2.5% and 97.5% points in the prior distribution. For many branches, the central 95% of the posterior distributions do not overlap providing clear evidence of compositional heterogeneity in the data.

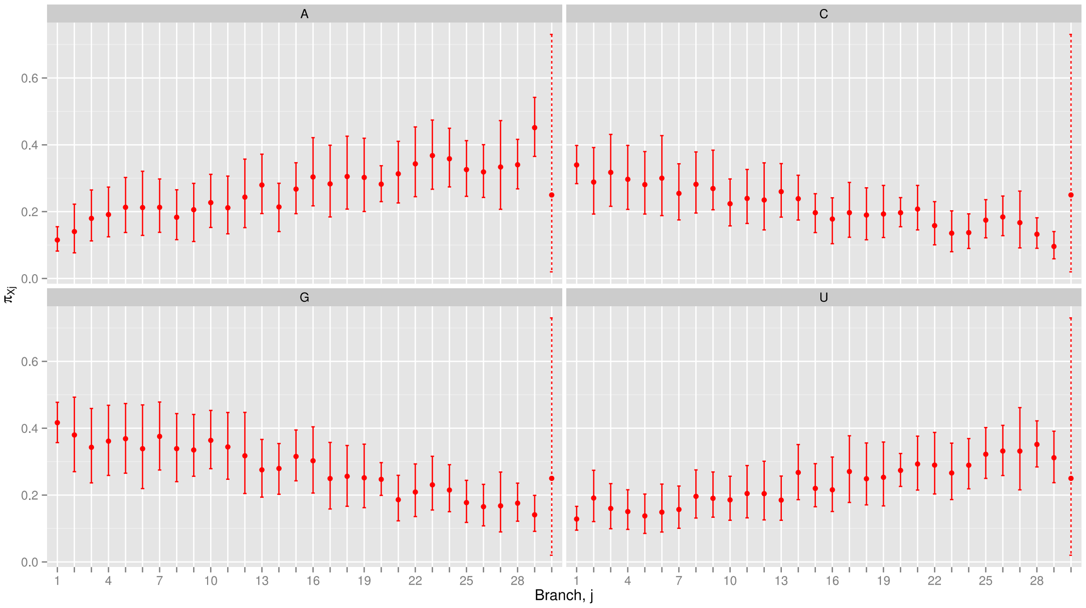

Supplementary Figure 2: Posterior inferences of nucleotide composition on each branch of the consensus tree of life inferred under the HB model. The posterior summaries are based on the consensus tree and branch labels in Figure 2(b) of the main text. See the caption for Supplementary Figure 1 for further details.

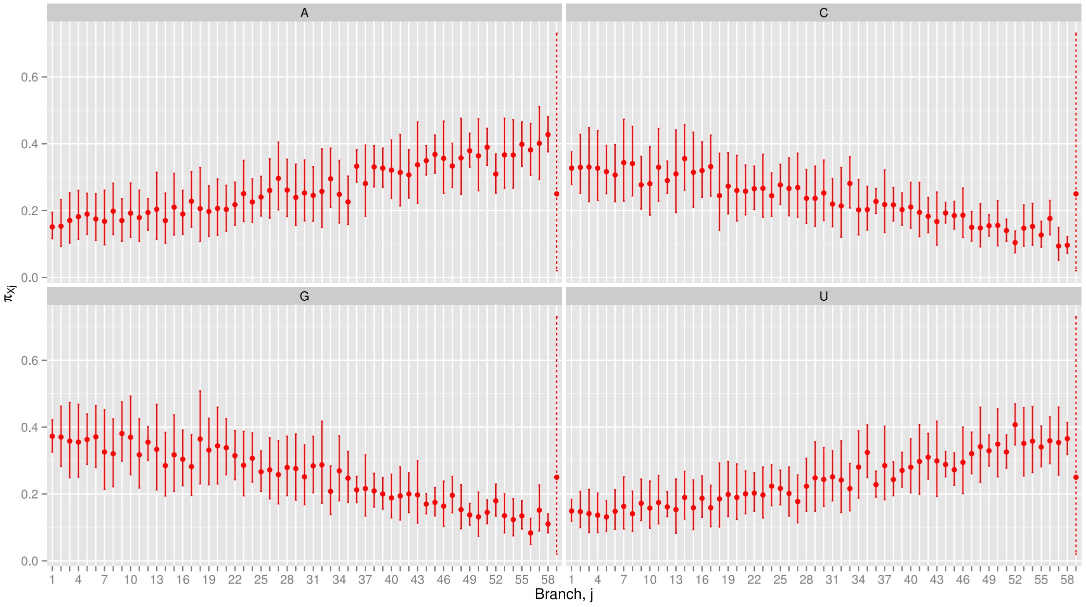

Supplementary Figure 3: Posterior inferences of nucleotide composition on each branch of the archaeal consensus tree inferred under the HB model. The posterior summaries are based on the consensus tree and branch labels in Figure 4 of the main text. See the caption for Supplementary Figure 1 for further details.

Supplementary Tables

Supplementary Table 1: Root splits receiving posterior support of at least 0.05 for the *Thermus-Deinococcus* dataset under the NR and HB models. The root partitions the taxa into two sets that we call the *root split*. We estimated the posterior probability for each root split as the proportion of trees in the MCMC sample which contained that root split. For both models, the split that received the most support separated *Aquifex* from the other taxa.

Posterior probability	Root split [smaller partition]
**NR Model**	
0.2139	[*Aquifex*]
0.1333	[*Bacillus*, *Deinococcus*]
0.1323	[*Bacillus*]
0.1055	[*Thermus*]
**HB Model**	
0.7006	[*Aquifex*]
0.1431	[*Thermotoga*]
0.0826	[*Deinococcus*, *Thermus*]
0.0576	[*Aquifex*, *Thermotoga*]

Supplementary Table 2: Root splits receiving posterior support of at least 0.05 for the tree of life dataset under the NR and HB models. The modal root split under the NR model separates Bacteria on one side from the Archaea and eukaryotes on the other. Under the HB model, the posterior for root splits is less concentrated, offering support to a variety of root positions within the Bacteria.

Posterior probability	Root split [smaller partition]
**NR Model**	
0.7031	[*Campylobacter jejuni*, *Chlamydia trachomatis*, *Clostridium acetobutylicum*, *Escherichia coli*, *Rhodopirellula baltica*, *Synechocystis sp.*]
0.0883	[*Rhodopirellula baltica*]
0.0708	[*Campylobacter jejuni*, *Chlamydia trachomatis*, *Clostridium*

	acetobutylicum, *Escherichia coli*, *Synechocystis sp.*]
**HB Model**	
0.3365	[*Campylobacter jejuni*, *Chlamydia trachomatis*, *Clostridium acetobutylicum*, *Escherichia coli*, *Synechocystis sp.*]
0.2197	[*Clostridium acetobutylicum*, *Synechocystis sp.*]
0.1765	[*Synechocystis sp.*]
0.1145	[*Rhodopirellula baltica*]
0.1009	[*Campylobacter jejuni*, *Chlamydia trachomatis*, *Escherichia coli*]

Supplementary Table 3: Root splits receiving posterior support of at least 0.05 for the Archaea dataset under the HB model. The posterior for root splits is fairly diffuse. The root in the consensus tree in Figure 4 receives support PP = 0.13 with a similar split, which places *Nanoarchaeum equitans* in the larger partition, receiving support PP = 0.07.

Posterior probability	Root split [smaller partition]
0.3161	[*Korarchaeum cryptofilum*]
0.1349	[*Methanocaldococcus jannaschii, Methanopyrus kandleri*, *Methanothermobacter thermautotrophicus*, *Pyrococcus furiosus*, *Thermococcus kodakarensis*]
0.1329	[*Aeropyrum pernix, Caldiarchaeum subterraneum*, *Caldivirga maquilingensis, Cenarchaeum symbiosum, Geoarchaeota NAG1, Hyperthermus butylicus*, *Ignicoccus hospitalis*, *Korarchaeum cryptofilum*, *Nanoarchaeum equitans, Nitrosopumilus maritimus, Pyrobaculum aerophilum, Staphylothermus marinus, Sulfolobus solfataricus, Thermofilum pendens*]
0.0726	[*Aeropyrum pernix, Caldiarchaeum subterraneum*, *Caldivirga maquilingensis, Cenarchaeum symbiosum, Geoarchaeota NAG1, Hyperthermus butylicus*, *Ignicoccus hospitalis*, *Korarchaeum cryptofilum*, *Nitrosopumilus maritimus*, *Pyrobaculum aerophilum, Staphylothermus marinus*, *Sulfolobus solfataricus*, *Thermofilum pendens*]
0.0667	[*Methanopyrus kandleri*]

0.0502	[*Aeropyrum pernix*, *Caldivirga maquilingensis*, *Geoarchaeota NAG1*, *Hyperthermus butylicus*, *Ignicoccus hospitalis*, *Pyrobaculum aerophilum*, *Staphylothermus marinus*, *Sulfolobus solfataricus*, *Thermofilum pendens*]

Supplementary Table 4: Posterior inferences of across-branch compositional heterogeneity in the *Thermus*, tree of life, and Archaea datasets. Values correspond to the standard deviations of proportions of each of the four nucleotides on each branch of the tree under the HB model. The archaeal dataset shows significantly more compositional variation than the other datasets.

**Dataset**	A	G	C	T
Thermus	0.0205	0.0198	0.0261	0.0257
Tree of life	0.0237	0.0267	0.0254	0.029
Archaea	0.0454	0.064	0.0486	0.0346

1. Heaps, S. E., Nye, T. M. W., Boys, R. J., Williams, T. a & Embley, T. M. 2014 Bayesian modelling of compositional heterogeneity in molecular phylogenetics. *Stat. Appl. Genet. Mol. Biol.* , 1–21. (doi:10.1515/sagmb-2013-0077)

2. Steel, M. & McKenzie, A. 2001 Properties of phylogenetic trees generated by yule-type speciation models. *Math. Biosci.* **170**, 91–112. (doi:10.1016/S0025-5564(00)00061-4)

3. Cherlin, S., Nye, T. M. W., Boys, R. J., Heaps, S. E., Williams T. A. & Embley T. M. 2015 The effect of non-reversibility on inferring rooted phylogenies. arXiv: 1505.08009.[q-bio.PE]
